# Validation of the Women’s Views of Birth Labor Satisfaction Questionnaire (WOMBLSQ4) in the Spanish Population

**DOI:** 10.3390/ijerph17155582

**Published:** 2020-08-02

**Authors:** María Dolores Pozo-Cano, Adelina Martín-Salvador, María Ángeles Pérez-Morente, Encarnación Martínez-García, Juan de Dios Luna del Castillo, María Gázquez-López, Rafael Fernández-Castillo, Inmaculada García-García

**Affiliations:** 1 Faculty of Health Sciences, University of Granada, 18071 Granada, Spain; pozocano@ugr.es (M.D.P.-C.); emartinez@ugr.es (E.M.-G.); rafaelfernandez@ugr.es (R.F.-C.); igarcia@ugr.es (I.G.-G.); 2 Faculty of Health Sciences, University of Granada, 52005 Melilla, Spain; 3 Faculty of Health Sciences, University of Jaen, 23071 Jaen, Spain; 4 Faculty of Medicine, University of Granada, 18071 Granada, Spain; jdluna@ugr.es; 5 Faculty of Health Sciences, University of Granada, 51001 Ceuta, Spain; mgazquez@ugr.es

**Keywords:** validation study, satisfaction questionnaire, birth attention, patient satisfaction

## Abstract

The satisfaction of women with the birth experience has implications for the health and wellness of the women themselves and also of their newborn baby. The objectives of this study were to determine the factor structure of the Women’s Views of Birth Labor Satisfaction Questionnaire (WOMBLSQ4) questionnaire on satisfaction with the attention received during birth delivery in Spanish women and to compare the level of satisfaction of pregnant women during the birth process with that in other studies that validated this instrument. A cross-sectional study using a self-completed questionnaire of 385 Spanish-speaking puerperal women who gave birth in the Public University Hospitals of Granada (Spain) was conducted. An exploratory factor analysis of the WOMBLSQ4 questionnaire was performed to identify the best fit model. Those items that showed commonalities higher than 0.50 were kept in the questionnaire. Using the principal components method, nine factors with eigenvalues greater than one were extracted after merging pain-related factors into a single item. These factors explain 90% of the global variance, indicating the high internal consistency of the full scale. In the model resulting from the WOMBLSQ4 questionnaire, its nine dimensions measure the levels of satisfaction of puerperal women with childbirth care. Average scores somewhat higher than those of the original questionnaire and close to those achieved in the study carried out in Madrid (Spain) were obtained. In clinical practice, this scale may be relevant for measuring the levels of satisfaction during childbirth of Spanish-speaking women.

## 1. Introduction

The birth of a child is one of the most significant events in the lives of women and their families. Knowing the level of satisfaction regarding the care received during the birth and postpartum periods is of special interest as it may help to improve the quality of health systems [1]. In Western countries, these experiences are becoming less frequent due to the drop in birth rates observed in many of them, especially in southern European countries, and it is hoped that the birth experience can become as rewarding as possible, despite not being free of serious consequences for the health of women and their newborns [1,2,3].

Many authors have explained the importance of women’s satisfaction with the birth process, because it influences such important aspects as the maintenance of breastfeeding [4], which is crucial for the health of mothers and newborns [5,6].

When women experience unsatisfactory or traumatic births, their memories will be of pain, anger, fear, or sadness, and they may even suffer from post-traumatic stress disorders or may not remember anything about the delivery process [7,8,9]. Furthermore, a bad experience in a previous delivery increases the anxiety and fear in subsequent deliveries [10,11]. The proximity of childbirth activates memories of previous traumatic experiences and abuse as well as psychiatric disorders in women that can trigger a fear of vaginal childbirth and increase the demand for caesarean births, thus increasing the risks to maternal and perinatal health [12].

This is why, at present, the perceived satisfaction regarding care received during the birth is considered an essential indicator to measure quality of care [13]. Hodnett describes the personal expectations of pregnant women, the support and quality of the relationship with health professionals, especially midwives, and the participation of women in decision making as the most influential elements [14].

There are various instruments that measure the satisfaction of women with childbirth [2,15,16,17,18,19,20,21,22]. The Women’s Views of Birth Labor Satisfaction Questionnaire (WOMBLSQ4) [23] has been used extensively in the recent literature and identifies women’s satisfaction with their birth labor and delivery experiences, as well as the pain relief received during and after. It was developed in the United Kingdom by Smith and has been translated into French and validated to be applied to French-speaking women in University Hospitals in Geneva (Switzerland), and into Spanish, where Marín-Morales et al. did the same with women who gave birth in hospitals in Madrid (Spain) [23,24,25].

In the Autonomous Community of Andalusia (Spain), the Public Health System is committed to achieving excellence in healthcare. This is understood as a comprehensive concept involving multiple variables, among which citizen satisfaction is an inalienable element [26]. Birth care is focused on women, providing them with personalized care and promoting their autonomy and their role in decision-making [27].

The version translated into Spanish also presents discrepancies in the number of factors with respect to the original version and its translation into French due to significant convergence problems that make it necessary to eliminate the “control” factor, thus leaving the scale in nine dimensions. In order to assess the satisfaction of Andalusian mothers in the process of birth labor and to check the structure of the instrument, the WOMBLSQ4 scale was translated into Spanish and validated.

The objectives of this study are to determine the factor structure of the WOMBLSQ4 questionnaire on satisfaction with the care received during birth in Spanish women and to compare the level of satisfaction of pregnant women during the birth process with other studies that validated this instrument.

## 2. Materials and Methods

### 2.1. Sample and Data Collection

A cross-sectional study was carried out between January and March 2019 in puerperal women who had given birth in the Public University Hospitals of the city of Granada (Spain). In the year prior, an average of 5000 deliveries had taken place at both hospitals. Through intentional sampling, 385 Spanish-speaking puerperal women aged 18 years old or older were selected by collaborating with midwives in the studio. The included women voluntarily agreed to participate and signed an informed consent self-completed questionnaire that was delivered in a sealed envelope and later collected by the principal investigator. Those who did not understand Spanish and had elective caesarean births were excluded.

Postpartum surveys were administered to 450 women, of whom 15 refused to complete them, 40 did not deliver babies, and 10 did not provide informed consent. The questionnaires that were complete for all items were considered valid. The final sample consisted of 385 women, which constitutes a response rate of 85.5%.

### 2.2. Materials

To evaluate women’s satisfaction with care received during delivery, the final version of the WOMBLSQ4 scale was used, which consists of 32 questions with Likert-type responses and 10 dimensions: professional support during the birth (5 questions), expectations of delivery (4 questions), assessment at home at the beginning of birth labor (3 questions), first contact with the newborn (3 questions), support of the husband/partner during labor (3 questions), pain relief during labor (3 questions), pain relief immediately after delivery (3 questions), continuity (2 questions), environment during delivery (2 questions), and control (2 questions). The measure of general satisfaction involved two questions [23]. The factorial validity of the scale was confirmed, as well as an adequate global reliability (Cronbach’s alpha 0.89), and the validity of the subscales was also shown (Cronbach’s alpha values ranged between 0.62 and 0.91). The score for each dimension was obtained by adding the values obtained in each question (some of them with an inverse score), and later on, the result was transformed so that the minimum possible score was 0 and the maximum possible one was 100 (total satisfaction in the dimension) [23,24,25]. Higher scores indicated greater satisfaction on the part of the women.

For this research, the scale was translated into Spanish by two English language translators and its final content was agreed upon by three midwives with extensive experience in childbirth assistance. The translated questionnaire was piloted to 50 women, and it was demonstrated that the instrument presented an excellent level of comprehension and an adequate completion time since, when collecting it, the participants were asked if they had difficulty completing it, if they understood all the questions, and if it seemed too long. 

In addition, the following sociodemographic variables were incorporated: age, marital status, educational level, and employment situation.

### 2.3. Data Analysis

A descriptive analysis was performed in which means and standard deviations were calculated for the quantitative variables and frequencies and percentages for the qualitative ones. The factorial structure of the scale was explored by extraction of the main components followed by a Varimax rotation. In the first analysis, the Kaiser–Meyer–Olkin (KMO) sample adequacy measure was calculated, accepting values greater than 0.70 as optimal measures. Subsequently, the Bartlett sphericity test was applied to show significant differences between the items in the correlation and the unit matrix.

Next, the communality of each of the items on the scale was studied, and those that showed values less than 0.30 were eliminated, as they were poorly represented in the factorial set obtained. Those factors with eigenvalues greater than 1 were considered, and the percentage of variance explained with the said factors was determined to assess the weight of each one. After the rotation and analysis of the item saturation table, these were assigned to the dimension in which their saturation was highest. Once the items were eliminated, the previous steps were repeated in order to obtain the final factor structure. The internal consistency of each of the subscales was measured using Cronbach’s alpha. Data analysis was performed with the SPSS v. Statistical package. 26.0 (International Busines Machines Corporation (IBM), Armonk, NY, USA) for Windows.

### 2.4. Ethical Considerations

The study complies with the standards of good clinical practice, explicit in the European Directive 2001/20/EC and Law 14/2007 (of 3 July) on biomedical research. The treatment of personal data in health research is governed by the provisions of the Organic Law 3/2018, 5 December, Protection of Personal Data and Guarantee of Digital Rights in Spain. The research protocol obtained a favorable resolution from the Ethics and Research Committee of Health Institutions.

## 3. Results

The sociodemographic characteristics of the analyzed sample are reflected in Table 1. The mean age of the participants was 31.62 years (SD 5.32), with a range of 18 to 46 years old. Regarding the level of education, almost half (175, 46.2%) had a university-level education. In relation to marital status, the majority were married or had a partner (359, 94.0%).

Regarding the labor situation, 184 (47.9%) were employed workers.

### 3.1. Exploratory Facial Analysis

To carry out the factor analysis, firstly, all items on the scale were considered, and a mean KMO sample adequacy of 0.80 was obtained, with the result of the Bartlett sphericity test being statistically significant (*p* < 0.001).

Of the 32 items in the original questionnaire, only three showed communalities below 0.50: 25 (I am satisfied with just one or two things about the labor care that I received: 0.441), 31 (I didn’t need a lot of pain relief after the birth: 0.475) and 12 (The way my labor care was provided could not have been improved: 0.476). However, they have not yet been removed from the questionnaire.

Using the main components method, nine factors that showed self-values greater than one, explaining 68.0% of the global variance, were extracted. A new dimension (3) was designed—pain during and after delivery—after merging dimensions six (pain during delivery) and seven (pain after delivery) from Smith’s original questionnaire [23].

Table 2 shows the Cronbach’s alpha and variance explained by each factor, as well as the saturations of each item, once the Varimax rotation had been performed.

Item 12 (the way my labor care was provided could not have been improved) showed a saturation of 0.55 and the generalization of its statement could be confusing. Item 25 did not saturate well with respect to the other two (0.46), and due to its statement (I am satisfied with just one or two things about labor care that I received), it did not seem to correspond to the being analyzed. Finally, both items were removed from the questionnaire.

Later on, a second analysis was performed with the remaining items, obtaining a sample adequacy of KMO of 0.86 and maintaining statistical significance in the Bartlett sphericity test (*p* < 0.001). This time, only items 3 and 31 showed communalities of less than 0.50, (0.44 and 0.47, respectively), although we decided to keep them in the model. The number of factors extracted by the principal component method with eigenvalues greater than 1 was also nine, which explained 70.0% of the global variance.

Table 3 represents the saturation level in the rotated components, the corresponding Cronbach’s alphas, and the variance explained by each factor.

In Table 3, the dimension of pain again appears to be merged. Item 31 (I didn’t need a lot of pain relief after the birth) has a saturation level close to 0.50 and continues to remain on the scale, although it is poorly associated with the other items, because its contents belong to this dimension.

Table 4 shows the Cronbach’s alpha values from the validation carried out in this study as well as those from the English version, the French adaptation, and the puerperal period in Madrid (Spain). It can be seen that the Cronbach’s alpha values of this study are in the range of previous studies or, in some cases, even higher.

### 3.2. Level of Satisfaction in the Different Versions

Table 5 shows the mean scores in each of the dimensions for the different versions. It can be seen that the three best valued dimensions in the four versions were professional support, support of the husband, and first contact with the newborn.

## 4. Discussion

The response rate was 85.0%, similar to that obtained in the study by Floris et al. [24] in Geneva, Switzerland, and somewhat higher than that obtained by Marín-Morales et al. [25] in Madrid (Spain).

The scale designed in this study showed a high validity and some good psychometric characteristics for measuring childbirth satisfaction in women based on their sociocultural environment.

It can be used in primiparous and/or multiparous women; pregnant women of low, medium, and high risk; puerperal women who have had vaginal births, whether spontaneous or instrumental; and even those who have had unscheduled caesarean sections. However, it is not applicable for women who have had scheduled (elective) caesarean births since, in most cases, these women would not be able to complete some items. In these circumstances, other dimensions not considered in the original scale should be considered.

The obtained percentage of women’s satisfaction with the care received during their births by factor analysis was somewhat lower than that shown by the original scale [23]; however, we understand that it is adequate for identifying those aspects that can be improved.

In relation to the first dimension, “Professional support”, the psychometric characteristics of our study showed slightly lower values than those of Smith [23], but higher than those achieved by Floris et al. [24] and Marín-Morales et al. [25]. This is the first factor identified in all of these studies and the one that best explains women’s satisfaction. It is made up of five items, all of them stated in a positive way, and results similar to those of previous studies were obtained [28,29,30,31]. All of them indicate that the kind and correct treatment of professionals and good communication favor the satisfaction of women, especially highlighting the role of the midwife as the professional who provides the most support during the delivery process, describing her as “competent”, “inspiring confidence”, or “wonderful” [32].

In the second dimension, “Expectation”, made up of four questions expressed in a positive way, the parameters obtained are similar to those obtained in the previous dimension. In their research, Melender et al. postulated that if the expectations of the pregnant women are in accordance with their lived experiences during childbirth, their evaluation of childbirth will be satisfactory [33]. Many women look for information on the sensations that they may experience during the labor process and idealize how it should go. In preparation for childbirth sessions the expectations created, information from other mothers, previous experiences, and the signals of their own bodies influence the elaboration of a mental image of delivery [34]. In other cases, despite experiences of severe pain or complications during a previous delivery that are different from their expectations, women feel motivated and encouraged to have another child due to having received good support from the midwife during the process [32].

The third dimension, “Pain during and after childbirth”, integrates two dimensions of the original version, which also appears in the French version. Our results coincide with a study carried out on Spanish women from Madrid (Spain). In both cases, the items were related to pain. In this new dimension, there are 2 items stated positively and 4 negatively, which coincides with the original and French versions. The reliability of the original version is superior to that of the other studies, while the results of this investigation are superior to the version carried out with women from the center of Madrid (Spain) and partially superior to the French version.

Item 31 “I didn’t need a lot of pain relief after the birth” is the only one of these new dimensions that has an adjusted value in our study. This is probably explained by the fact that most of the women in our sample received epidural analgesia for childbirth, and the effects of this analgesia remain in the immediate postpartum period [35,36,37]. This fact means that puerperal women do not need many analgesics in this period.

In the early puerperium period (from 3 h after birth to 10–15 days later), women may experience pain due to uterine involution, the presence of hemorrhoids, and even breast pain [38,39]. There may also be perineal pain following or without an episiotomy [39,40], but in most cases, pain relief is necessary. On the other hand, this item is not relevant for those women who wanted a natural childbirth and therefore would consider it unnecessary. Therefore, it could be a potentially upgradeable question.

The fourth dimension, “Home assessment”, presents a very similar Cronbach’s alpha value in the four versions, the highest of which corresponds to the original version. In all versions, with the exception of the version from Madrid (Spain), this dimension consists of three items written in negative form. The coefficients obtained in our study were slightly lower than those obtained by Smith [23], but higher than those found by Marín-Morales et al. [25].

In Spain, as in most countries of the Organization for Economic Cooperation and Development (OCEDE) [41], except in the Netherlands and the United Kingdom, there is no culture of maternal care at home [42,43]. In the Spanish Health System, both public and private, most pregnant women go to hospitals when they have their first contractions to be evaluated at the beginning of labor and births are completed in them. Anecdotally, births at home are attended by professionals from the private sector.

In relation to the fifth dimension, “Support from husband”, the psychometric parameters obtained are similar to those in the original version. Item 29 “I would have preferred to have more support from my partner/husband” is negative, and its value is the lowest on our scale. This factor identified in the analysis is a component of satisfaction that, in our environment, is favored by legislation [44]. Along the line of humanization of perinatal care, in 1995, in the Autonomous Community of Andalusia, the right of pregnant women to be accompanied by a person they trust during the prepartum, delivery, and postpartum periods was legislated [45]. This legal support for the figure of the companion has been highly valued in numerous studies [30,32,46].

The sixth dimension, “Holding baby”, consists of two negative and one positive item, and the psychometric characteristics found in our study have somewhat higher values than those obtained in Madrid (Spain) [25]. Early contact with the newborn, in addition to being an indicator of women’s satisfaction, favors the establishment of an emotional bond between mother and child [47]. The search for greater prominence, that is granted to women in our Public Health System through the implementation of the Childbirth and Birth Plan of the Autonomous Community of Andalusia, provides mothers with the possibility of expressing their preferences during birth as the right to have their son or daughter by their side during the hospital stay [46,48]. Whenever the state of the newborn and the mother allow it, skin-to-skin contact between the two should be promoted, since it provides benefits to both the mother and the newborn: maintenance of a good body temperature, an increase in blood glucose levels, and helps to maintain breastfeeding and weight [49,50,51,52].

The seventh dimension, “Knowledge of women by professionals during birth assistance”, showed an adequate Cronbach’s alpha value. The title of this dimension is formulated in the same sense as the French version and both differently from the original version. The content of the questions is focused on the continuity raised by Australian authors [53,54] and adapted to the title formulated in our research.

The values of the parameters obtained in the eighth dimension, “Environment”, are adequate and most of them are superior to those collected the study of the Center of Spain [25]. This dimension consists of two questions and one of them is negative. An environment that facilitates intimacy, silence, environmental warmth, and the absence of medicalized furniture contributes to the satisfaction of women [55,56]. However, in the qualitative study carried out by Jenkins et al. in the state of New South Wales (Australia), most women did not highlight the environment as one of the three most important aspects in their care [53].

“Control” was the ninth dimension and showed a great relationship with the satisfaction of women and their experience in childbirth. Various authors have pointed out that perceived control over the situation increases satisfaction; this is a dimension that has been widely incorporated in Anglo-Saxon research [53,55,57,58,59,60]. However, in our study, similarly to that of Smith and Floris et al., this dimension was the last to be shown as a factor and also the one that explained the smallest percentage of satisfaction [23,24]. Probably for this reason, this dimension was excluded in the version carried out by Marín-Morales et al. with women from Centre of Spain [25]. This result shows that, despite the fact that, in recent years, it has been an incentive by the State and Health Institutions for women and families to take control and responsibility over their health through the inclusion of the Autonomy Law of the patient (2002), the change in the healthcare model based on paternalism has prevailed for so many years in our healthcare system that it is not yet something that the population considers to be of special importance [61].

The two questions about General Satisfaction, number 12 (the care during the delivery process could not have been better) and number 25, (I am satisfied with only one or two things about the care I received during the delivery process) were not analyzed as in the original study. Both questions, especially the last one, are so general that they suggest a certain ambiguity, since it is difficult to assess satisfaction with one or two of these aspects. However, the resulting mean scores are mostly somewhat higher than those obtained by Smith [23] in the original version, although they are similar to those obtained by Marín-Morales et al. [25] in their study carried out in Madrid (Spain).

We understand that, at present, childbirth satisfaction questionnaires should incorporate the control dimension, since, in the current context, the empowerment of women and decision-making during childbirth is a priority in healthcare [62].

### 4.1. Limitations

Given the good understanding of the scale items and the good adaptation of the scale, it would have been interesting to have expanded the sample to other Spanish-speaking areas.

### 4.2. Recommendations for Future Research

It would be advisable for future research to merge dimensions 6 “pain relief during childbirth” and 7 “pain relief immediately after childbirth” into one dimension, as shown in the original version. Both dimensions are related to pain relief.

In this dimension, there is item 31, which asks about the need to relieve pain immediately after delivery. We believe that this question should be changed, as many analgesics are not necessary immediately after delivery, only if in the postpartum period.

## 5. Conclusions

In the resulting model from the WOMBLSQ4 questionnaire, the nine dimensions measure the level of satisfaction with childbirth care in puerperal women. The resulting average scores are mostly somewhat higher than those obtained in the original version, and close to those achieved in the study carried out in Madrid (Spain). In clinical practice, this scale is relevant for measuring satisfaction levels in Spanish-speaking women.

## Figures and Tables

**Table 1:** Sociodemographic characteristics of the sample.

Socio-Demographic Variables		
	**x ± SD**	**Range**
**Age**	31.6 ± 5.32	18–46
	***n***	**%**
**Level of Education (*n* = 379)**		
University	175	46.2
Vocational training	87	23.0
Secondary education	66	17.4
Primary/Elementary/Basic education	51	13.5
**Marital Status (*n* =382)**		
Married or with a partner	359	94.0
Single	23	6.0
**Labor Situation (*n* = 384)**		
Employed workers	184	47.9
Housewives	68	17.7
Busines women	26	6.8
Unemployed	92	24.0
Other work circumstances (studying, retired, etc.)	14	3.6

**Table 2:** Analysis of each dimension and items on the scale.

1 Professional Support (Cronbach’sAlpha = 0.867, % Variance Explained = 14.02)		Coefficient*
**Q19**	During labor there was always a carer to explain things so that I could understand.	0.843
**Q7**	All my labor carers were very supportive.	0.834
**Q13**	Carers always listened very, very carefully to everything that I had to say.	0.808
**Q27**	All my carers treated me in the most friendly and courteous manner possible.	0.778
**Q32**	My carers couldn’t have been more helpful.	0.733
**Q12**	The way my labor care was provided could not have been improved.	0.556
**2 Expectations (Cronbach’s Alpha = 0.861, % Variance Explained = 9.19)**		
**Q17**	The delivery went almost completely as I had hoped that it would	0.809
**Q11**	The labor went nearly exactly as I had hoped that it would.	0.794
**Q22**	My labor was just about the right length.	0.719
**Q1**	My labor went totally normally.	0.710
**3 Pain During and After the Birth (Cronbach’s Alpha = 0.781, % Variance Explained = 8.75)**		
**Q26**	More pain relief would have made my labor easier. (−)	0.719
**Q6**	I should have been offered something more to relieve the pain I had after my baby was born. (−)	0.702
**Q16**	I was in a fair bit of pain immediately after the birth. (−)	0.669
**Q9**	I should have been offered something more to relieve my labor pains. (−)	0.668
**Q20**	I got excellent pain relief in labor.	0.586
**Q31**	I didn’t need a lot of pain relief after the birth.	0.399
**4 Home Assessment (Cronbach’s Alpha = 0.843, % Variance Explained = 7.52)**		
**Q15**	When I thought that my labor had started, I would have liked a carer to come and see me at home to confirm that I had. (−)	0.914
**Q28**	Early home assessment of me in labor would have been very helpful. (−)	0.904
**Q8**	I should have had a home assessment in early labor. (−)	0.761
**5 Support from Husband (Cronbach’s Alpha = 0.750, % Variance Explained = 6.80)**		
**Q2**	My birth partner/husband helped me to understand what was going on when I was in labor.	0.937
**Q23**	My birth partner/husband couldn’t have supported me any better.	0.920
**Q29**	I could have had a bit more help from my birth partner/husband. (−)	0.511
**6 Holding Baby (Cronbach’s Alpha = 0.675, % Variance Explained = 6.74)**		
**Q18**	I needed to hold my baby a little earlier than I did. (−)	0.842
**Q10**	After my baby was born, I was not given him/her quite as soon as I wanted. (−)	0.786
**Q3**	I got to see my baby at exactly the right time after she/he was born.	0.577
**7 Knowledge of Women about Professionals During Childbirth Assistance** **(Cronbach’s Alpha = 0.797, % Variance Explained = 5.21)**		
**Q24**	I knew the carer(s) present at the birth of my baby.	0.855
**Q5**	At the start of my labor I knew my carers very well.	0.844
**8 Environment (Cronbach’s Alpha = 0.711, % Variance Explained = 4.97)**		
**Q4**	My birth room was a little impersonal and clinical. (−)	0.810
**Q14**	The area where I gave birth was very pleasant and relaxing.	0.764
**9 Control (Cronbach’s Alpha = 0.436, % Variance Explained = 4.76)**		
**Q21**	Everyone seemed to tell me what to do in labor. (−)	0.753
**Q30**	Labor was just a matter of doing what I was told by my carers. (−)	0.729
**Q25**	I am satisfied with just one or two things about the labor care that I received. (−)	0.460

* Correlation coefficients of each item with its subscale.

**Table 3:** Analysis of each dimension and items on the scale after the removal of items Q12 and Q25.

1 Professional Support (Cronbach’s Alpha = 0.869, % Variance Explained = 13.403)		Coefficient*
**Q19**	During labor there was always a carer to explain things so that I could understand.	0.836
**Q7**	All my labor carers were very supportive.	0.830
**Q13**	Carers always listened very, very carefully to everything that I had to say.	0.801
**Q27**	All my carers treated me in the most friendly and courteous manner posible.	0.772
**Q32**	My carers couldn’t have been more helpful.	0.722
**2 Expectations (Cronbach’s Alpha = 0.861, % Variance Explained = 9.817)**		
**Q17**	The delivery went almost completely as I had hoped that it would.	0.819
**Q11**	The labor went nearly exactly as I had hoped that it would.	0.808
**Q22**	My labor was just about the right length.	0.725
**Q1**	My labor went totally normally.	0.719
**3 Pain during and after the Birth (Cronbach’s Alpha = 0.749, % Variance Explained = 9.085)**		
**Q6**	I should have been offered something more to relieve the pains I had after my baby was born. (−)	0.717
**Q26**	More pain relief would have made my labor easier. (−)	0.716
**Q16**	I was in a fair bit of pain immediately after the birth. (−)	0.682
**Q9**	I should have been offered something more to relieve my labor pains. (−)	0.660
**Q20**	I got excellent pain relief in labor.	0.575
**Q31**	I didn’t need a lot of pain relief after the birth.	0.418
**4 Home Assessment (Cronbach’s Alpha = 0.843, % Variance Explained = 8.026)**		
**Q15**	When I thought that my labor had started, I would have liked a carer to come and see me at home to confirm that I had. (−)	0.912
**Q28**	Early home assessment of me in labor would have been very helpful. (−)	0.903
**Q8**	I should have had a home assessment in early labor. (−)	0.762
**5 Support from Husband (Cronbach’s Alpha = 0.750, % Variance Explained = 7.209)**		
**Q2**	My birth partner/husband helped me to understand what was going on when I was in labor.	0.940
**Q23**	My birth partner/husband couldn’t have supported me any better.	0.927
**Q29**	I could have had a bit more help from my birth partner/husband. (−)	0.498
**6 Holding Baby (Cronbach’s Alpha = 0.675, % Variance Explained = 7.042)**		
**Q18**	I needed to hold my baby a little earlier than I did. (−)	0.842
**Q10**	After my baby was born, I was not given him/her quite as soon as I wanted. (−)	0.784
**Q3**	I got to see my baby at exactly the right time after she/he was born.	0.579
**7 Knowledge of Women about Professionals during Childbirth Assistance (Cronbach’s Alpha = 0.797, % Variance Explained = 5.532)**		
**Q24**	I knew the carer(s) present at the birth of my baby.	0.855
**Q5**	At the start of my labor I knew my carers very well.	0.847
**8 Environment (Cronbach’s Alpha = 0.711, % Variance Explained = 5.297)**		
**Q4**	My birth room was a little impersonal and clinical. (−)	0.834
**Q14**	The area where I gave birth was very pleasant and relaxing.	0.771
**9 Control (Cronbach’s Alpha = 0.481, % Variance Explained = 4.646)**		
**Q30**	Labor was just a matter of doing what I was told by my carers. (−)	0.789
**Q21**	Everyone seemed to tell me what to do in labor. (−)	0.778

* Correlation coefficients of each item with its subscale.

**Table 4:** Cronbach’s alpha values from the different studies analyzed.

	V. Granada (Spanish)	V. Original (English)	V. Geneva (French)	V. Madrid (Spanish)
	C.’s Alpha	C.’s Alpha	C.’s Alpha	C.’s Alpha
1 Professional support	0.869	0.91	0.84	0.74
2 Expectations	0.861	0.90	0.86	0.80
3 Pain in labor and Pain after the birth	0.749	0.83 & 0.65	0.79 & 0.59	0.68
4 Home assessment	0.843	0.90	0.87	0.83
5 Support from husband	0.750	0.83	0.56	0.61
6 Holding baby	0.675	0.87	0.78	0.51
7 Knowledge of women about their caretakers during the birth	0.797	0.82	0.84	0.36
8 Environment	0.711	0.80	0.67	0.43
9 Control	0.481	0.62	0.53	-----
10 General satisfaction	0.421	0.75	0.85	-----

**Table 5:** Average scores in the different versions.

Dimensions	V. Granada (Spanish)	V. Original (English)	V. Geneva (French)	V. Madrid (Spanish)
	Mean ± SD	Mean ± SD	Mean ± SD	Mean ± SD
1 Professional support	83.71 ± 12.4	72.3 ± 20.1	80.9 ± 19.5	91.35 ± 12.9
2 Expectations	64.16 ± 20.8	59.0 ± 27.7	64.2 ± 29.7	60.88 ± 29.8
3 Pain in labor and Pain after delivery	65.59 ± 27.9	60.8 ± 23.5	65.15 ± 27.0	64.98 ± 22.9
4 Home assessment	60.16 ± 17.9	54.3 ± 20.5	64.6 ± 24.7	69.31 ± 30.0
5 Support from husband	80.42 ± 25.5	72.7 ± 21.2	75.2 ± 21.0	90.48 ± 15.9
6 Holding baby	78.09 ± 25.5	74.2 ± 21.8	78.1 ± 25.7	82.61 ± 25.8
7 Knowledge of women about their caretakers during the birth	51.75 ± 23.2	38.8 ± 21.0	38.0 ± 30.2	67.49 ± 27.7
8 Environment	56.74 ± 19.8	61.6 ± 28.2	59.6 ± 27.4	40.18 ± 22.2
9 Control	49.31 ± 21.2	53 ± 23.7	46.5 ± 27.1	-----
10 General satisfaction	70.11 ± 18.3	53.1 ± 22.2	66.5 ± 13.5	83.33 ± 22.2
